# The impact of “Child Care” intervention in rural Primary Health Care Program on prevalence of diarrhea among children less than 36 months of age in rural western China

**DOI:** 10.1186/s12887-018-1172-1

**Published:** 2018-07-11

**Authors:** Wenlong Gao, Guirong Li, Xiaoning Liu, Hong Yan

**Affiliations:** 1 Department of Epidemiology and Health Statistics, School of Public Health, Health Science Center, Xi’an Jiaotong University, Xi’an, Shaanxi 710061 People’s Republic of China; 2 Institute of Epidemiology and Health Statistics, School of Public Health, Lanzhou University, Lanzhou, Gansu People’s Republic of China; 3 Department of Pediatrics, Gansu Provincial Maternal and Child Care Hospital, Lanzhou, Gansu People’s Republic of China

**Keywords:** “Child Care” intervention: CCI, Rural Primary Health Care, Diarrhea, Children less than 36 months of age

## Abstract

**Background:**

It was unclear how and to what extent the “Child Care” intervention (CCI) in rural Primary Health Care Program affected the prevalence of childhood diarrhea in rural western China.

**Methods:**

The available data of 10,829 and 10,682 households was collected from shared 34 counties of 9 provinces of western China in 2001 and 2005 respectively. A log-binomial regression model was used to predict the effect of CCI on prevalence of childhood diarrhea.

**Results:**

In 2001, the prevalence rate of diarrhea among children less than 36 months of age was 17.01% in intervention group and 17.72% in control group, and in 2005 this crude rate declined to 4.85% in the former and 6.84% in the latter. Log-binomial regression analysis showed that CCI decreased the overall prevalence of childhood diarrhea by 27% (adjusted relative prevalence ratio (*r*PR) = 0.73 95% CI 0.59, 0.89). The stratification regression by social-economic status (SES) of the households showed that this effect varied with SES of the households. In the medium or rich households, this intervention was effective significantly (the medium: adjusted *r*PR = 0.63,95%CI 0.41,0.95; the rich: adjusted *r*PR = 0.72,95%CI 0.54,0.97), but in poor households it seemed to be less effective (adjusted *r*PR = 0.86,95%CI 0.55,1.36).

**Conclusion:**

In rural Primary Health Care Program, CCI was effective in improving childhood diarrhea but this effect was inequitable among SES of the households. So, attention should be paid to the inequality when CCI was adopted to reduce childhood diarrhea in rural China.

**Electronic supplementary material:**

The online version of this article (10.1186/s12887-018-1172-1) contains supplementary material, which is available to authorized users.

## Background

Millennium Development Goals (MDGs) 4 was declared to have reduced the under-5 mortality rate by two-thirds between 1990 and 2015 [1]. Reaching the MDG4 will require universal coverage with key effective, affordable interventions and priority actions, including promotion, prevention, and care for mothers and children [2]. Those diseases with the highest burden such as childhood diarrhea became a major concern in the process of achieving the goal. The Rural Primary Health Care (RPHC) program as the main governmental effort launched by Chinese Ministry of Health and the United Nations Children’s Fund attempts to promote the utilization of health care services and improve primary health care through some comprehensive targeted interventions to accelerate the realization of MDG in rural western China. In the Rural Primary Health Care (RPHC) program for rural western China from 2001 to 2005, some specific interventions such as “Child Care” intervention (CCI), “Safe Motherhood” intervention and “Rational Drug Use” intervention were implemented. Of these interventions, only CCI was a child-oriented group intervention.

Though deaths caused by childhood diarrhea had declined substantially since the 1980s, diarrhea was still one of the leading causes of under-5 mortality in developing countries and thus continued to remain the most main public health concern especially in those under-developing regions [3–5]. Lowering the prevalence of childhood diarrhea to reduce diarrheal mortality will be of critical importance for improving child survival and health. Though the RPHC program had been implemented twice (the first round:1999 to 2000; the second round: 2001 to 2005) in rural western China, it was unclear how and to what extent CCI included in the second round RPHC program affected the prevalence of childhood diarrhea in these areas.

In China, no study attempted to evaluate the impact of CCI on prevalence of diarrhea among children less than 36 months of age in rural western China. The objective of this study is to highlight the effect of child-oriented interventions in the second round RPHC program on childhood diarrhea, which may give some insights to the development of a reasonable strategy of diarrhea prevention and control among children less than 36 months of age in rural western China.

## Methods

### Subjects and settings

Two cross-sectional surveys of the second round RPHC program were conducted in 2001 and 2005 respectively. The baseline survey in 2001 involved 46 counties of 9 western provinces (Gansu, Guangxi, Jiangxi, Inner Mongolia, Ningxia, Qinghai, Sichuan, Xinjiang and Chongqing) and the final survey in 2005 included 45 counties of 10 provinces (the above-stated 9 plus Guizhou province). In both surveys, all the counties, not sampled randomly, were pre-assigned by the Chinese Ministry of Health and UNICEF. Other sample units such as the townships, the villages and households were obtained with a probability-proportion-to-size sampling method (PPS). The specific sampling procedure went as follows: five townships were selected out of each county, four villages out of each sampled township and sixteen households with children less than 36 months of age out of the selected villages with a completely random sampling method. If there had been more than 16 households in one village, only 16 were selected randomly; if there had been less than 16, all the households were selected and the rest ones were selected from the neighboring villages. In all the sampled households, if more than one child was present, the one child was randomly selected, and his/her caretaker was interviewed face-to-face with a pre-code family questionnaire by trained professional interviewers after they had signed the informed consent form. The family questionnaires involved the information related to a family’s socio-demographic characteristics, child care and maternal prenatal health care. In the surveyed items on child care, the information of childhood diarrhea in the previous two weeks at the time of the survey was obtained. In both surveys, diarrhea was defined as the passage of three or more loose or watery stools in the preceding 24 h. After the survey, the body length and weight of the child were measured.

### Intervention measures

CCI was an important measure for growth and development of children in the second round RPHC program. It was implemented as follows: 1) Measuring body length and weight for growth monitoring, screening low birth weight and developing the reasonable feeding guidance purposefully for children less than 36 months of age; 2) Carrying out community nutrition intervention through township hospitals’ guiding the village clinics, extensive publicity and family guidance; 3) General administration of vitamin A twice a year, covering more than 90% of children aged 6–36 months and making 0.6 million children aged 6–36 months benefit from this invention; 4) Vaccinating Neonates against hepatitis B virus and preventing vertical transmission of hepatitis B. All these targeted interventions in the second round RPHC program were implemented through the three-tie county-township-village rural health care network to each targeted household from 2001 to 2005. In every village of the intervened areas, the professional personnel were responsible for the implementation of the intervention measures to every household with children aged 6–36 months.

Of all the surveyed counties in 2001 and 2005, 34 were shared. Of these shared counties, 9, which accepted CCI, were classified as intervention group and 25, which did not accept CCI, as control group. All the rest were excluded from the study.

### Main study variables

The outcome variable of interest in the study was two-week prevalence of childhood diarrhea. Socio-economic status (SES) of families was assessed by the demographic and health survey wealth index generated with the five variables (type of vehicle, water supply, income resource, texture of pot and type of used salt). After making an analysis of a principal component for the above-mentioned 5 variables by year, the first principal component was stored into the dataset. According to the tertiles of the first principal component, the socioeconomic status of the families was classified into three categories: poor, medium and rich. The variables of intervention and survey year were considered as main effect measures. The nutritional status of these children was assessed with age-specific height Z score (HAZ), age-specific weight Z score (WAZ) and weight-specific height Z score (WHZ). Those children with HAZ, WAZ or WHZ less than − 2 were identified as stunting, underweight or wasting respectively. But all extreme values were excluded (HAZ below − 6 or above + 6, WAZ below − 6 or above + 5, and WHZ below − 5 or above + 5) [6]. Other factors such as family size, child size, education and age of mothers, ethnicity, drinking boiled water, sex and age of the child, birth site and way of delivering a child were also identified as some possible potential confounders to adjust the predictors of the effect of the intervention on prevalence of childhood diarrhea.

### Statistical analysis

In the study, the effect of CCI on prevalence of childhood diarrhea was a result of combined action of the two factors of intervention and year so the interaction effect between the intervention and survey year variables is estimated. In fact, this interaction effect can be expressed as the ratio of the intervention-to-control prevalence ratio (*PR*) of childhood diarrhea in 2005 to that in 2001, which here is named as relative prevalence ratio (*rPR*) in order to distinguish it from the main effect of intervention. (eg, *rPR* = *PR*_2005_/*PR*_2001_). When dependent variable is set to childhood diarrhea and intervention variable, year variable, and their interaction item as well as other adjusted variables were entered together into this regression model, log-binomial regression model can obtain the logarithm of *rPR* for the intervention through the coefficient of the interaction item [7, 8]. The equation of log-binomial regression model can be expressed as follows:

$$ \log P\left(Y=1\left|\mathit{\operatorname{int}}, year,X\right.\right)=\alpha +\beta year+\gamma int+\varphi year\times \mathit{\operatorname{int}}+\omega X $$

Here, *P*(*Y* = 1|*int*, *year*, *X*) is the prevalence rate of childhood diarrhea; *int*, *year* and *X* represent the intervention, survey year and adjusted variables respectively. *α* is a constant; *β* and *γ*are the main effects of survey year and intervention variables respectively. From the equation, *φ* is just the estimator of logarithmic *rPR* for the intervention and its confidence interval can be obtained from the model. Therefore, *rPR* is equal to *e*^*φ*^.

SPSS 17v software (SPSS Inc., Chicago, IL, USA) was used to analyze all data in the study. Chi-square test was used to compare the proportions between different categories and test the trend of decline in prevalence of diarrhea by year. All observed factors including SES, family size, child size, drinking boiled water, age of mother, maternal education, ethnicity, age and gender of child, township-or-above level delivery and natural delivery, and intervention variable, year variable and their interaction were entered together into a log-binomial regression model to predict the effect of CCI on prevalence of childhood diarrhea. In addition, the log-binomial regression models were also used to assess the change of the important indexes in CCI such as vitamin A supplementation (VAS), nutritional status and breastfeeding between intervention and control groups while the five factors (age and sex of children, age of mothers, maternal education and ethnicity) were adjusted. When the log-binomial regression models were non-convergent, COPY method was used to estimate the effect of CCI [9]. The significant test level was set for 0.05.

## Results

### Sample characteristics and other related factors

In 2001, the control group had 7936 households with the children less than 36 months of age and the intervention one had 2893. All data of the study are included in Additional file 1. In 2005, the control group had 7885 and the intervention one had 2797. Table 1 showed the socio-demographic characteristics and other related factors of the surveyed households by intervention in 2001 and 2005. Except for sex of children, other factors were unbalanced in varying degrees between intervention and control groups in 2001 or 2005.

**Table 1:** other related factors and indices of the surveyed households by intervention and year

	2001		2005	
	Control	Intervention	Control	Intervention
SES				
Poor	2558(32.2)	501(17.3) ^*c*^	2728(34.7)	491(17.6) ^*c*^
Medium	2804(35.3)	930(32.2)	2424(30.8)	611(21.9)
Rich	2574(32.4)	1462(50.5)	2718(34.5)	1693(60.6)
Family size (>5)	4398(55.4)	925(32.0) ^*c*^	4547 (57.7)	1215(43.4) ^c^
Child size (one)	4782(60.3)	1996(69.0)^*c*^	4350(55.2)	1898(67.9) ^*c*^
Drinking boiled water	6324(79.7)	2011(69.5)^*c*^	7148(90.7)	2547(91.1)
Age of mother (Mean, SD)	26.51(4.0)	26.98(4.0) ^*c*^	27.20(4.8)	27.09(4.4)
Maternal education (>9y)	538(6.8)	252(8.7) ^*b*^	559(7.1)	251(9.0) ^*b*^
Ethnicity(Han)	5690(71.7)	1972(68.2)^*c*^	5677(72.0)	1918(68.6) ^*c*^
Age of children (m)				
0–11	2713(34.2)	975(33.7)	2645(33.5)	1196(42.8) ^*c*^
12–23	2984(37.6)	1071(37.0)	2916(37.0)	1045(37.4)
24–36	2239(28.2)	847(29.3)	2324(29.5)	556(19.9)
Boy	4508(56.8)	1693(58.5)	4493(58.0)	1642(58.7)
Breastfeed status	7575(95.6)	2707(93.3)	7542(95.9)	2626(94.0) ^*c*^
Nutritional status				
Stunting	1616(20.9)	534(19.2)	911(12.5)	287(10.3) ^*b*^
Underweight	1258(16.3)	532(19.1) ^*c*^	502(6.8)	183(6.6)
Wasting	306(4.0)	135(4.8) ^*a*^	417(5.7)	134(4.8)
VAS in the last year	1992(25.1)	241(8.3) ^*c*^	5538(70.2)	1634(58.4) ^*c*^
Township-or-above level delivery	4172(52.6)	1075(37.2)^*c*^	6902(87.5)	2505(89.6) ^*b*^
Natural delivery	7120(89.7)	2639(91.2)^*a*^	6536(82.9)	2365(84.6) ^*a*^

Intervention vs control: *P*^*a*^<0.05, *P*^*b*^<0.01, *P*^*c*^<0.001; SES: socio-economic status; SD: standard deviation; VAS: vitamin A supplementation

Use of oral vitamin A in the previous year, nutritional status and breatfeeding status are three key indecies in CCI. In 2001, utilization rate of oral vitamin A in control group was 25.1% and that in intervention group was 8.3%, but in 2005, the rate increased to 70.2 and 58.4% respectively. Overall, childhood under-nutritional status was also obviously improved over time. The prevalence of stunting and underweight in intervention groups decreased by 8.9 and 12.5% respectively and in control groups by 8.4 and 9.5% respectively. The prevalence of wasting in control groups seemed to have a conspicuous rise but that in intervention groups remained unchanged. Though the breastfeeding in both intervention groups and control groups kept a very very high prevalence, its prevalence after CCI seemed to have fallen a little bit.

When sex and age of children, age of mothers, maternal education and ethnicity were adjusted, the results of log-binomial regression showed that *PR* of VAS was increased by almost 2-folds (r*PR* = 2.99 95%CI:2.63,3.39); that *PR* of stunting was decreased by 7% (r*PR* = 0.93, 95%CI 0.85,0.998); that CCI seemed to be less contributive to the prevalence of underweight significantly (r*PR* = 0.89, 95%CI 0.74,1.07) but wasting had a 30% improvement in *PR* (r*PR* = 0.70, 95%CI 0.53,0.92); that CCI also may take no effect on breastfeeding (*rPR* = 1.00,95%CI 0.98,1.01).

### Diarrhea prevalence in intervention and control group

**Fig. 1:**
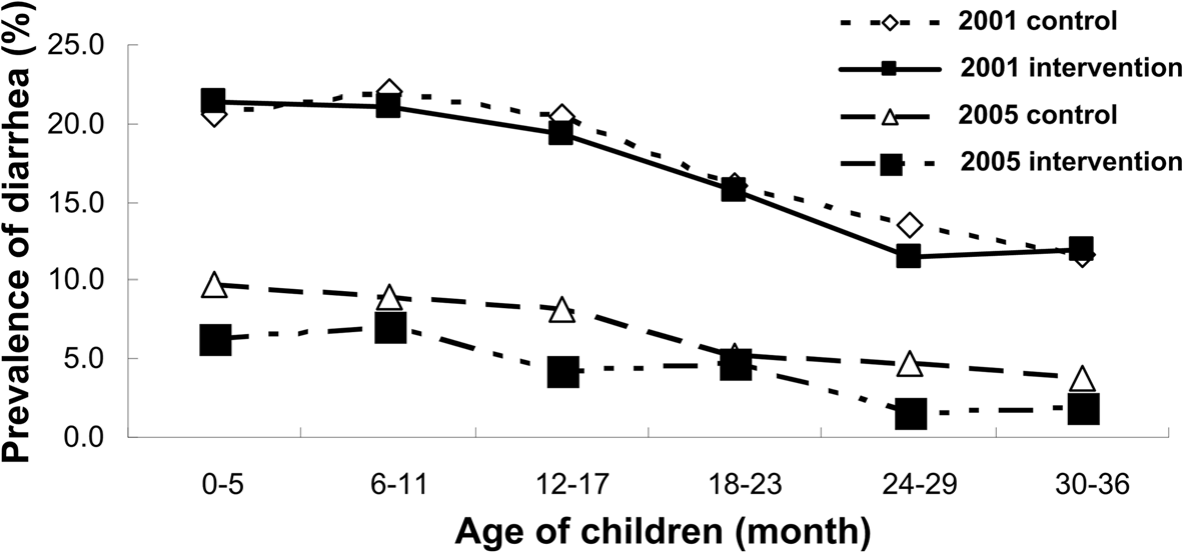
The age-specific prevalence of childhood diarrhea by year and intervention

**Fig. 2:**
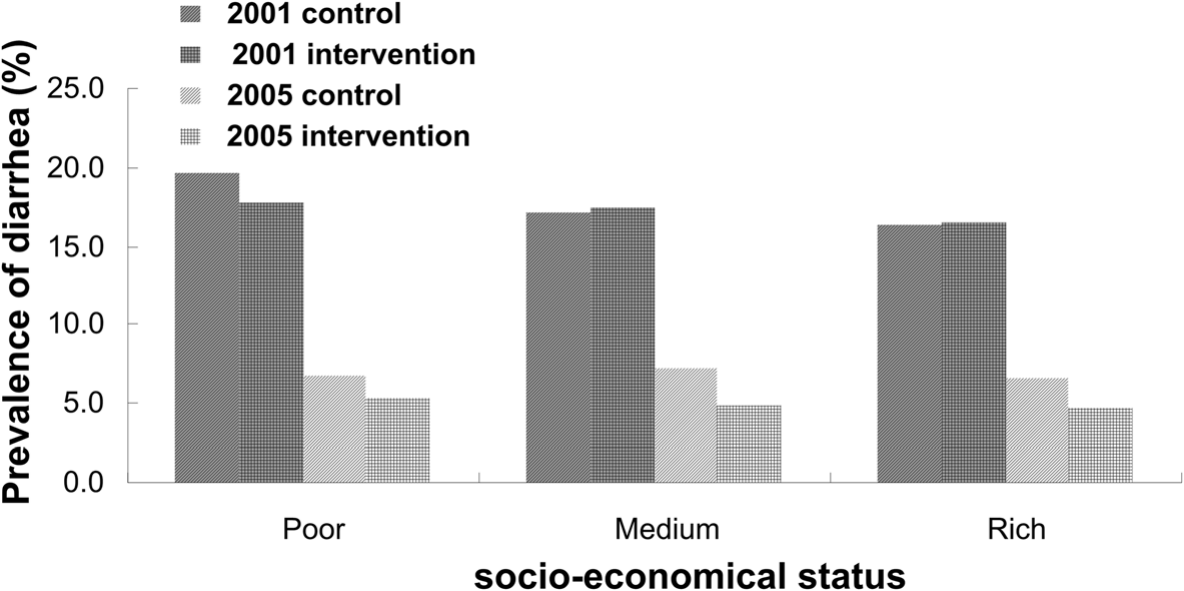
The SES-specific prevalence of childhood diarrhea by year and intervention

In 2001, the prevalence rate of diarrhea among children less than 36 months of age was 17.01% (95%CI:15.63~ 18.37%) in intervention groups and 17.72% (95%CI:16.86~ 18.54%) in the control groups, and in 2005, this crude rate declined to 4.85% (95%CI:4.10~ 5.70%) and 6.84% (95%CI:6.24~ 7.36%) respectively. Figure 1 shows the age-specific prevalence of childhood diarrhea by year and intervention: whatever age group, the prevalence of diarrhea in 2005 was lower than that in 2001 and the overall tendency in prevalence of diarrhea declined gradually with the age in all situations. Figure 2 depicts the SES-specific prevalence of childhood diarrhea by year and intervention. In 2001 or 2005, the prevalence of childhood diarrhea was falling with the improvement of SES, which can be observed in either control or intervention group, but only in control group of 2001, the declining trend of diarrhea prevalence with improvement of SES was significant statistically (*×*^2^ = 11.04, *P* = 0.004).

### Impact of CCI on prevalence of childhood diarrhea

Table 2 shows the impact of CCI on prevalence of diarrhea among children less than 36 months of age by SES. When the potential confounders were controlled for, CCI could make the overall prevalence of childhood diarrhea decreased by 27% (adjusted *r*PR = 0.73 95%CI 0.59, 0.89). The stratification regression by SES showed that the effect of this intervention varied with SES of the households. In the medium or rich households, it could make the prevalence of childhood diarrhea decreased significantly (the medium:adjusted *rPR* = 0.63,95%CI 0.41,0.95;the rich: adjusted *rPR* = 0.72,95%CI 0.54,0.97) but in poor households, the significant effect was not observed (adjusted *rPR* = 0.86,95%CI 0.55,1.36).

**Table 2:** the effect of CCI on prevalence of childhood diarrhea by SES of households

	the unadjusted effect of CCI		the adjusted effect of CCI	
	r*PR*	95% CI	r*PR*	95% CI
^*a*^Overall	0.74	0.60–0.91	0.73	0.59–0.89
^*b*^SES				
Poor	0.87	0.56-1.36	0.86	0.55–1.36
Medium	0.67	0.44–1.01	0.63	0.41–0.95
Rich	0.71	0.53–0.95	0.72	0.54–0.97

*CCI* “Child Care” Intervention, *SES* Socio-economic Status, *rPR* relative prevalence ratio, *CI* confidence interval

^a^Eleven variables (SES, family size, child size, drinking boiled water, age of mother, maternal education, ethnicity, age and gender of child, township-or-above level delivery and natural delivery), and intervention variable, year variable and their interaction were entered together into a log-binomial regression model to evaluate the adjusted effect of CCI on childhood diarrhea

^b^Except for SES, other variables were entered together into a log-binomial regression model to evaluate the adjusted effect of CCI on childhood diarrhea

## Discussion

In western China, poor sanitary condition and health service status led to the high morbidity and mortality of childhood diarrhea. So, it was the first priority of the Chinese government in the process of accelerating the achievement of Millennium Development Goal 4 of reducing childhood mortality [10]. In our study, a downward trend of childhood diarrhea prevalence over time was apparent. A change that prevalence of childhood diarrhea in intervention groups (a 12.2% reduction) decreased from 2001 to 2005 could also be observed in the control group (a 10.9% reduction). During the study, the water and sanitation infrastructure in these rural areas, which made the prevalence of diarrhea severely decreased, had been greatly improved and the interventions of water, sanitation and hygiene had facilitated the access of clean water [11]. These regional sanitary measures taken to solve the countryside problems of water and hygiene could account for the parallelled decrease in prevalence of childhood diarrhea not only in intervention groups but also in control ones. In addition, our recent study aiming at explaining the prevalence of childhood diarrhea and its decrease in rural western China highlighted the importance of a set of social and economic determinants that were considered in the current study to be potential confounders of the CCI effect [12].

When those potential confounders were controlled for, the log-binomial regression analysis shows that CCI could decrease the overall prevalence of childhood diarrhea by 27%. The reduced effect directly benefited from achievement of some important intervention indicators of CCI such as VAS and the improvement of childhood nutritional status. In our study, the *PR* of VAS in the intervention groups increased by 2-folds compared with that in the control groups. Some studies had demonstrated that VAS could decrease the severity, duration and prevalence of childhood diarrhea [13–15]. Certainly, it needs to be clarified, as shown by baseline survey, that the prevalence of VAS in the control groups was higher than that in the intervention groups (control vs intervention: 25.1% vs 8.3%). In fact, from 1999 to 2000, the first round of RPHC program was implemented in 40 counties of 5 provinces (Gansu, Xinjiang, Ningxia, Qinghai and Guizhou) [16]. All these provinces but Guizhou were included in the current study and all their counties entered into the control groups of the current study were covered by the first round of the program. But, all those counties in the intervention group were not covered by the first round of the program. The regional impact of the first round program (1999–2000) could reasonably account for the difference of VAS baseline level between intervention and control groups. In addition, many previous studies found that childhood under-nutrition especially stunting was associated with childhood diarrhea [17, 18]. A substantial reduction in prevalence of childhood stunting was observed clearly in our study: *PR* of stunting after CCI had a 7% drop compared with that of stunting before CCI and the risk of wasting prevalence also was decreased by CCI. Though in our study underweight after CCI did not have a significant improvement in prevalence, it had a substantive limitation in evaluting childhood undernutrition in that weight-for-age is difficult to discriminate temporary and permanent undernutrition [19].To our knowledge, the improvement of childhood under-nutrition, which promoted the development of children’ body and then increased their disease-resistant ability and enhanced their immunity, reduced the susceptibility of diarrhea. As for breastfeeding, CCI seemed to take no effect yet. The improvement of VAS, stunting and wasting included in CCI led to the substantial reduction in prevalence of childhood diarrhea.

Our study also found that the impact of CCI on prevalence of childhood diarrhea varied with SES of the households. In the medium or rich households, the effect of reducing prevalence of childhood diarrhea was significant but in poor households it seemed to become less effective. This phenomenon implied a socio-economic inequality in improvement of childhood diarrhea by CCI. A similar phenomenon had also been observed in improving other childhood health problems [19, 20]. All program and policy makers seemed to have expected an actual reduction of diverse inequities in health status between rich and poor groups through intervention [21]. However, CCI in our study seemed to have widen rather than narrowed the gap in prevalence of childhood diarrhea between poor and rich households, which met the “inverse equity hypothesis” [21]. Certainly, the inequality can be reduced through more equitable distribution of resources led by fairer social and economic policy [22]. Thus, a new resource-allocated policy should be made to improve the socio-economic inequality in households and oversee in the whole process of implementation of the intervention.

The strength of the current study is that it highlights the impact of CCI on prevalence of diarrhea among children less than 36 months of age by means of the data of the population-based large sample and to supply some policy implications of the interventions for childhood diarrhea.

The limitation of the current study should be acknowledged. First, county selection was not random, and the intervention and control groups were not randomly allocated. As a result, the evaluated effect may be subject to unobserved confounding factors. Then, all data were collected on the basis of caretakers’ recall so recall bias seemed to be inevitable. Finally, other unobserved factors such as duration and severity of diarrhea and so on were not included in our study, which might affect the evaluated effect.

## Conclusion

In conclusion, “Child Care” intervention strategies decreased the prevalence of childhood diarrhea significantly and this effect was inequitable among SES of the households. So, attention should be paid to the inequality when CCI was adopted to reduce childhood diarrhea in rural China.

## Additional file

Additional file 1: Data of the study. (CSV 2181 kb)

## Abbreviations

CCI: “Child Care” Intervention;
HAZ: age-specific height Z score;
MDGs: Millennium Development Goals;
PPS: a probability-proportion-to-size sampling method
*PR*: prevalence ratio;
RPHC: Rural Primary Health Care;
*rPR*: relative prevalence ratio;
SES: social-economic status;
VAS: vitamin A supplementation;
WAZ: age-specific weight Z score;
WHZ: weight-specific height Z score;
